# Public Health collapse during floods in Pakistan: A call for National & Global Response

**DOI:** 10.12669/pjms.41.10.13355

**Published:** 2025-10

**Authors:** Ayesha Fahim, Habib Ahmad Qureshi, Ahsan Sethi

**Affiliations:** 1 Ayesha Fahim, Islamic International Dental College, Riphah International University, Pakistan. The Equator University of Science and Technology, Uganda; 2 Habib Ahmad Qureshi, College of Medicine, King Faisal University, Alahsa, Saudi Arabia; 3 Ahsan Sethi, Department of Public Health, College of Health Sciences, QU Health, Qatar University, Doha, Qatar

The July–August 2025 monsoon season inflicted catastrophic damage across Pakistan, destroying 8,975 homes, damaging over 660 kilometers of roads, and killing more than 6,000 livestock. These losses exacerbated displacement, malnutrition, and access barriers to healthcare.^1^ As of 13 September 2025, 964 lives were lost, 1181 were injured, and more than 4.3 million people had been displaced.^1^ While current official data from provincial health departments remain limited, patterns from the 2022 floods underscore the likely scale of impact: over 7,000 cholera cases and 4,500 dengue infections were reported in Sindh alone during a comparable three-week period.^2^ In the current response, 14,322 individuals have been treated across various relief camps, though no treatment data has yet been reported from Sindh, despite significant disease exposure.^1^ The risk of large-scale outbreaks remains high amid widespread water contamination, food insecurity, and inadequate sanitation.^3^ The collapse of sanitation systems, marked by latrine destruction, contaminated wells, and widespread open defecation, has triggered sharp increases in diarrhoeal and skin infections across multiple flood-hit districts. Regions including Sindh, southern Punjab, and Khyber Pakhtunkhwa have reported rising cases of acute fever, malaria, skin infections, and diarrhoeal illness with the onset of the monsoon season.^1^ Previous flood crises in Pakistan also revealed critical disruption in antenatal care, chronic disease management, and childhood immunization services, patterns likely to recur under current operational constraints.^2^ Pregnant and lactating women face heightened vulnerability due to disrupted maternal care, while the absence of gender-sensitive sanitation and psychosocial services has placed adolescent girls at increased risk of trauma and gender-based violence. Displaced children in Swat, Battagram, and Lower Dir lack access to child-safe spaces or psychosocial support services, exposing them to trauma, distress, and exploitative environments.^3^ In districts such as Shangla and Battagram, health infrastructure damage and critical medicine shortages have rendered basic care inaccessible, compounding disease risk and disrupting primary services.^3^ Reports from the 2025 floods confirm a dire humanitarian crisis, with specific concerns raised about malnutrition among children in displacement camps lacking consistent access to food and clean water.^3^ Disrupted supply chains and rising food prices have further intensified food insecurity for displaced families. Mental health support was largely absent, particularly for displaced women and adolescent girls, compounding psychosocial trauma and gender-based risks. 3 School closures and the conversion of classrooms into shelters deprived children of education and safe spaces, exacerbating long-term psychosocial stress.

Despite these overwhelming circumstances, frontline healthcare workers, including lady health workers, mobile clinic teams, and volunteers from the Pakistan Red Crescent, National Disaster Management Authority (NDMA), and Pakistan Armed Forces, have been central to maintaining access to basic care, often working in flooded conditions with limited supplies. NDMA, through partner organisations like the World Health Organisation, established over 1200 relief camps providing basic health facilities including medicines, vaccinations, hygiene kits, and water purification tablets in high-burden districts.^1^ Emergency services conducted 1,879 rescue operations, evacuating over 502,000 individuals, with the highest burden in Punjab.^1^ In several districts, frontline health providers operated without electricity or cold chain systems, compromising vaccine storage and essential medication supply. Moreover, real-time data reporting remained inconsistent due to damaged communication infrastructure, limiting rapid needs assessment and delaying targeted responses.^4^ These efforts illustrate that Pakistan’s health response, though constrained, was not absent.

The scale and recurrence of such disasters align with global projections. The Intergovernmental Panel on Climate Change (IPCC) Sixth Assessment Report warns that South Asia is among the most at-risk regions from intensified flooding, with health systems facing disproportionate strain.^5^ The Lancet Countdown on Health and Climate Change has likewise highlighted Pakistan as a frontline state where climate-driven health risks are rising faster than its adaptive capacity.^6^ Despite being among the countries most vulnerable to climate shocks, Pakistan has not fully implemented a nationwide health disaster response framework. The absence of a flood-triggered epidemic surveillance system shows how lessons from the 2022 floods were not institutionalized. Despite widespread displacement, over 800 deaths, and the destruction of nearly 5,000 homes, the government has yet to declare a national emergency, reflecting institutional under recognition of the growing intersection between climate shocks and public health disasters.^3^ The National Disaster Management Authority (NDMA) focused primarily on infrastructure rehabilitation, while the National Institute of Health (NIH) and provincial health departments lack surge capacity for epidemic surveillance, emergency medical logistics, or adaptive service delivery.^7^ There are no flood-triggered epidemic surveillance protocols, centralized medical logistics dashboards, or mobile health deployment strategies.^8^ The absence of a coordinated public health emergency plan, especially within displacement camps, results in preventable illness and avoidable mortality.^9^ Progress on infrastructure restoration has not been matched by investment in resilient public health capacity. International relief efforts have also been slow and fragmented, with donor fatigue leaving many communities without essential support.^10^, ^11^ Beneath the immediate destruction lies a more protracted crisis, the collapse of public health capacity under climate pressure. While government and partner agencies demonstrated capacity for emergency mobilization, especially in rescue operations and camp establishment, the absence of long-term investment in epidemic surveillance and resilient primary care left millions vulnerable to preventable disease.

Pakistan is responsible for less than 1% of global carbon emissions, but it suffers disproportionately from its impact. The global health community must treat Pakistan’s crisis as a warning signal and show solidarity by moving beyond promises to action. Equitable distribution of the Loss and Damage Fund agreed at COP27 is essential to supporting climate-vulnerable nations like Pakistan.^12^ Without accelerated financial mechanisms to support climate change adaptation efforts and climate-resilient health planning, similar collapses will increasingly occur across all vulnerable countries. There is a need for sharing early warning systems and related technologies with developing countries to better predict, prepare, and respond to extreme climate conditions. We urge global health actors and climate financiers to prioritize anticipatory public health investments, including real-time surveillance systems, disaster-resilient primary care infrastructure, medicine stockpiles, technology, and mobile service units equipped for emergencies. Moreover, international agencies should provide urgent humanitarian assistance, including food, shelter, clean water and medical care for millions displaced by the floods. International lenders should consider debt restructuring to enable climate-vulnerable countries like Pakistan to rebuild in the aftermath of a disaster. Building on the bravery of frontline health workers and the partial successes of 2025’s relief operations, Pakistan now needs structural reforms to ensure that resilience, not improvisation, becomes the default public health response. If seasonal flooding is becoming the norm, adaptive public health systems must follow. Remember, the cost of inaction at this stage will be paid in human lives.
